# Genome sequence of *Providencia rettgeri* ZAPR22R, isolated from calla lily in China

**DOI:** 10.1128/mra.01049-24

**Published:** 2025-01-29

**Authors:** Yin Jiang, Zhen Zeng, Zengxian Wang, Yi Wang, Rongxin Gou, Xiaorong Huang, Yushan Lu, Guojun Zhang, Zunzheng Wei

**Affiliations:** 1 College of Horticultural Science & Technology, Hebei Key Laboratory of Horticultural Germplasm Excavation and Innovative Utilization/Hebei Higher Institute Application Technology Research and Development Center of Horticultural Plant Biological Breeding, Hebei Normal University of Science and Technology, Qinhuangdao, China; 2 Institute of Grassland, Flowers and Ecology, Beijing Academy of Agriculture and Forestry Sciences, Beijing, China; 3 Taiyuan Municipal Construction Group Co., Ltd., Taiyuan, China; The University of Arizona, Tucson, Arizona, USA

**Keywords:** *Providencia rettgeri*, calla lily, genome

## Abstract

Here, we present the complete genome sequence of *Providencia rettgeri* strain ZAPR22R, isolated from the petiole and tuber of calla lily (*Zantedeschia hybrida*), infected with soft rot. The genome consists of a single chromosome (4,528,722 bp) with a G+C content of 41.1%.

## ANNOUNCEMENT

*Providencia rettgeri*, an opportunistic pathogen, has been described to be implicated in various diseases such as urinary tract infections, diarrhea, and meningitis (1). It can be isolated from diseased tissues, and genomic analysis indicates that *P. rettgeri* is resistant to several antibiotics (2). Additionally, *P. rettger*i has been proven to be one of the pathogens causing soft rot (3), which causes the decay of plant organs or tissues, and is fatal and difficult to control, resulting in substantial economic losses. The bacterial genome was obtained on the basis of our previous research.

*P. rettgeri* ZAPR22R was primordially isolated from the petiole and tuber of soft rot calla lily in Yanqing District, Beijing, China, in August 2022, in our previous work (3). Petiole and tuber samples were collected, surface-sterilized with 75% alcohol, and then rinsed with sterile distilled water (SDW) multiple times. The clean samples were then ground into a homogenate, plated onto Luria-Bertani (LB) agar, and incubated at 28°C for 12 h. Single colonies were selected for purification, and the purified single colony was propagated for DNA (FastPure Bacteria DNA Isolation Mini Kit, Nanjing Vazyme Biotech Co., Ltd.) extraction and genomic sequencing.

Library preparation and genome sequencing were performed at Biomarker Technologies, according to the protocol provided by PacBio. PacBio sequencing technology uses SMRT chip as the sequencing carrier. In the nanopore inside the SMRT chip, DNA polymerase and template are combined and four colors are fluorescently labeled with four bases (deoxyribonucleoside triphosphates [dNTPs]). In the base pairing stage, different bases are added, different light will be emitted, and the type of base can be judged according to the wavelength and peak of light. The DNA sample was sheared using g-TUBE, and then damage repair and terminal repair were performed. The dumbbell joints were connected, and the exonuclease digestion was carried out.The target fragments were screened using BluePippin, and then the sequencing libraries were obtained. Shorter reads (<2 kb) were filtered after sequencing using PacBio HIFI mode. Fastuniq v.1.1 (-t q), trimmomatic v.0.33 (LEADING:3 TRAILING:3 SLIDINGWINDOW:50:20 MINLEN:100), and filtlong v.0.2.0 were used for quality control. A total of 47,446 reads, with an average length of 8,635 bp, were obtained. The filtered subreads were assembled, followed by circularization (4). Spades v.3.6.2 (-m 300 -k 21,33,55,77,99,127 --cov-cutoff auto), canu v.1.5 (useGrid=false overlapper=mhap utgRe Align=true), wtdbg v.2.5, flye v.2.8.2, hifiasm v.0.12, racon v.1.4.0 (-u -t 4), and pilon v.1.22 (--mindepth 1 --changes --fix all) were used for assembly. Circlator v.1.5.5 (minimus2 --no_pre_merge) was used for ring judgment. Default parameters were used for all software unless otherwise specified. The genome of *P. rettgeri* ZAPR22R presumably consists of a single chromosome of 4,528,722 bp and exhibits a G+C content of 41.1% (Table 1). The topology of the contig was predicted to be circular.

**TABLE 1:** Characteristics of *Providencia rettgeri* ZAPR22R

Characteristic	Result
Sequencing	
Raw reads	
No. of reads	49,033
Size (bp)	418,203,977
Clean reads	
No. of reads	47,446
Size (bp)	409,703,740
N50 (bp)	9,216
Average read length (bp)	8,635
Genome	
Assembly size (bp)	4,528,722
Contig	1
G + C content (%)	41.1
No. of coding genes	4,084
Total gene size (bp)	3,865,407
Average gene length (bp)	946
Total repetitive sequence length (bp)	13,203
Repetitive sequence content (%)	0.29
rRNA	8,7,7 (5S, 16S, 23S)
tRNA	82
CRISPR	4
Genomic island	4
Prophage	4
Gene cluster	2
Protein subcellular localization	
Signal peptide	392
Transmembrane protein	1,023
Secreted protein	311

The NCBI Prokaryotic Genome Annotation Pipeline v.5.3 was used for annotation of public assembly (5), while coding genes were predicted using Prodigal v.2.6.3 for analyses reported here (6). Repeat sequences were searched by RepeatMasker v.4.0.5 (7). Transfer RNA (tRNA) genes were predicted with tRNAscan-SE v.2.0. Ribosome RNA genes were predicted with Infernal v.1.1.3 (8–10). CRT v.1.2, CRISPR, genomic island, prophage, and gene cluster were separately predicted by IslandPath-DIMOB v.0.2, PhiSpy v.2.3, and antiSMASH v.5.0.0 (11–14). The transmembrane proteins were filtered by TMHMM. The secretory proteins were detected by SignalP; after transmembrane proteins were filtered by TMHMM, the candidate secretory proteins could be obtained (15, 16). Analysis results are listed in Table 1.

## Data Availability

This complete genome of *P. rettgeri* has been deposited in the NCBI GenBank under the accession number CP173172.1 and BioProject accession number PRJNA1182479. The version described in this manuscript is the first version. The raw reads have been submitted to the SRA under accession numbers SRR31671221 and SRR31671222.
